# Medications for attention-deficit/hyperactivity disorder in individuals with or without coexisting autism spectrum disorder: analysis of data from the Swedish prescribed drug register

**DOI:** 10.1186/s11689-020-09352-z

**Published:** 2020-12-23

**Authors:** Viktoria Johansson, Sven Sandin, Zheng Chang, Mark J. Taylor, Paul Lichtenstein, Brian M. D’Onofrio, Henrik Larsson, Clara Hellner, Linda Halldner

**Affiliations:** 1 Department of Medical Epidemiology and Biostatistics, Karolinska Institutet, PO Box 281, SE-171 77 Stockholm, Sweden; 2 Centre for Psychiatric Research, Department of Clinical Neuroscience, Karolinska Institutet & Stockholm Health Care Services, Stockholm County Council, Stockholm, Sweden; 3 Department of Psychiatry, Icahn Medical School at Mount Sinai, New York, USA; 4 Seaver Autism Center for Research and Treatment, Icahn School of Medicine at Mount Sinai, New York, USA; 5 Department of Psychological and Brain Sciences, Indiana University, Bloomington, Indiana USA; 6 School of Medical Sciences, Örebro University, Örebro, Sweden; 7 Department of Clinical Science, Child and Adolescent Psychiatry, Umeå University, Umeå, Sweden

## Abstract

**Background:**

Clinical studies found that medication for attention-deficit/hyperactivity disorder (ADHD) is effective in coexisting autism spectrum disorder (ASD), but current research is based on small clinical studies mainly performed on children or adolescents. We here use register data to examine if individuals with ADHD and coexisting ASD present differences in the prescribing patterns of ADHD medication when compared to individuals with pure ADHD.

**Methods:**

Data with information on filled prescriptions and diagnoses was retrieved from the Swedish Prescribed Drug Register and the National Patient Register. We identified 34,374 individuals with pure ADHD and 5012 individuals with ADHD and coexisting ASD, aged between 3 and 80 years. The first treatment episode with ADHD medications (≥ 2 filled prescriptions within 90 days) and daily doses of methylphenidate during a 3-year period was measured. Odds ratios (ORs) were calculated for the likelihood of being prescribed ADHD medication in individuals with and without ASD and Wilcoxon rank-sum test was used to compare group differences in dose per day.

**Results:**

Individuals with ADHD and coexisting ASD were less likely to start continuous treatment with ADHD medication (ADHD 80.5%; ADHD with ASD 76.2%; OR, 0.80; 95% confidence interval, 0.75-0.86), were less likely to be prescribed methylphenidate, and were more commonly prescribed second line treatments such as dexamphetamine, amphetamine, or modafinil. No group difference was observed for atomoxetine. In adults with ADHD and coexisting ASD, methylphenidate was prescribed in lower daily doses over three years as compared to individuals with pure ADHD.

**Conclusions:**

The findings indicate that there are differences in the medical treatment of individuals with or without ASD. If these differences are due to different medication responses in ASD or due to other factors such as clinicians’ perceptions of medication effects in patients with ASD, needs to be further studied.

**Supplementary Information:**

The online version contains supplementary material available at 10.1186/s11689-020-09352-z.

## Background

Attention-deficit/hyperactivity disorder (ADHD) is associated with extensive psychiatric comorbidity [1], including autism spectrum disorder (ASD) [2–4] and around 20–50% of individuals with ADHD are thought to meet the criteria for ASD [5]. Medication is today one of the cornerstones of ADHD treatment [6], with an increasing use of ADHD medications worldwide [7]. Commonly used medications include stimulants (e.g., methylphenidate or amphetamines) and non-stimulants (atomoxetine), and medications for ADHD have a well-documented efficacy on ADHD symptoms in children as well as in adults [8]. Further, there is evidence for better school performances [9], lower risk for motor vehicle accidents [10], and less physical injuries [11] in individuals who received ADHD medication [11].

Individuals with coexisting ADHD and ASD are more severely impaired on a group-level, for example, regarding adaptive functioning and executive control [12]. Due to the lack of efficient treatments for core symptoms of ASD, optimized treatment of any comorbidity should be of importance. Individuals with coexisting ADHD and ASD should according to present guidelines be offered the same medical choices as individuals with ADHD only [6]. Meta-analyses on available randomized clinical trials (RCTs) show that ADHD medications, e.g., methylphenidate [13], and atomoxetine [14] improve ADHD symptoms in children and adolescents with ASD. One recent clinical study on adults with ASD showed that methylphenidate was effective [15]. Yet, some studies showed that ADHD medications were less tolerated in children and adolescents with ASD [13], and individuals with ASD presented higher discontinuation rates due to side effects such as irritability [16]. Individuals with ASD were also more sensitive to side effects and tolerated lower medication doses [17]. Clinical experience includes case reports about need for higher, as well as lower, doses of ADHD medication in patients with coexisting ADHD and ASD.

Previous studies of ADHD medication in individuals with ASD are hampered by methodological problems, such as limited study samples with restricted length of the follow-up periods, typically only a few months. The length of follow-up is crucial, considering that ADHD medication is prescribed on a long-term basis [18]. Further, most studies are restricted to children and adolescents, and less is known about the medication effects in the increasing number of adults with coexisting ADHD and ASD. Studies on medication dose levels are also needed considering previous findings that individuals with ASD may not tolerate higher doses [17]. In addition, the treatment effects from methylphenidate or atomoxetine have been frequently studied, while few studies have focused on treatments with amphetamines in ASD [19]. Above all, there are no previous studies directly comparing the effects of ADHD medication between individuals with ADHD with or without coexisting ASD. For example, conclusions on efficacy and side effects were mainly drawn from RCTs performed on individuals diagnosed with either ADHD or ASD. Improved scientific evidence is required for the clinician to assess the advantages and disadvantages of using psychostimulants in individuals with ASD [19].

Observational pharmacoepidemiology studies, using data from health registers that cover all filled prescriptions, allows for analyses following prescribing patterns in larger groups, over extended time-periods, and in broader age-categories, and may be a valuable complement to results obtained from RCTs [20]. To our knowledge, no large-scale register studies have directly compared prescribing patterns for ADHD medications in individuals with ADHD with or without coexisting ASD. In the present study, we examined whether individuals with coexisting ADHD and ASD, despite recommendations on similar ADHD treatment, present different prescribing patterns when compared to individuals with ADHD only. Specifically, we aimed to study whether individuals with ADHD and coexisting ASD (a) are less likely to initiate continuous treatment with ADHD medications overall, (b) are more likely to fill prescriptions from second line treatment options (e.g., dexamphetamine or amphetamine), and (c) are dispensed lower or higher doses of ADHD medication.

## Methods

### Registers

The cohort was established by linking health care registers including the National Patient Register (NPR), with psychiatric diagnoses and dates with codes in accordance with International classification of diseases (ICD) from 1973 (hospitalizations) and 2001 (specialized out-patient care); the Total Population Register with information on year of birth on all residents in Sweden; and the Swedish Prescribed Drug Register (PDR), with information on all filled prescriptions from pharmacies in Sweden from July 1, 2005 (drug class according to the Anatomical Therapeutic Chemical [ATC] Classification System, number of items [tablets/capsules], doses, and dispensation dates, for details see Supplement). The filled prescriptions in the PDR provide information on type and amount of medication taken out from the pharmacy. Written instructions from individual doctors regarding indication or recommended doses for the prescription or information on prescriber’s name or clinic was not available. Ethical approval was granted by the Regional Ethics Review Board in Stockholm (Dnr: 2013/5:8).

### Study cohort

From NPR, we identified all individuals (children and adults) with two or more diagnoses of ADHD with the first diagnosis of ADHD (ICD-10, F90) appearing from July 1, 2005, and onwards, which matches the launch of the Swedish PDR, allowing us to capture new treatment periods. For flow-chart of the study inclusion, see Supplement Figure 1. For distribution of when the year of the first ADHD diagnosis appears in the NPR, see Supplemental Table 1. To be able to follow individuals’ new treatment periods during three years until the end of register data in December 31, 2013, we excluded individuals with their first ADHD diagnosis after January 1, 2011. Similarly, to follow filled prescriptions from the PDR during three years, individuals were excluded, if their first filled prescription for ADHD medication occurred after January 1, 2011. Individuals with a first ADHD or ASD-diagnosis registered before age three were excluded. Despite a wide age range for the first diagnosis of ASD and/or ADHD (see Table 1), we chose not to exclude individuals who received an ADHD and/or ASD diagnosis at an advanced age.

**Table 1:** Descriptive statistics of individuals with attention-deficit/hyperactivity disorder (ADHD) with or without coexisting autism spectrum disorder (ASD)

	ADHD without coexisting ASD	ADHD with coexisting ASD	
	Number of individuals (%)	Number of individuals (%)	***p*** value*
**Total number of individuals**	34,374	5012	
**Sex**			
Males	21,918 (63.8)	3395 (67.7)	
Females	12,456 (36.2)	1617 (32.3)	< 0.0001
**Year of birth**			
> 2000	2187 (6.4)	477 (9.5)	
1991-2000	14,571 (42.4)	2257 (45.0)	
1981-1990	7243 (21.1)	1202 (24.0)	
1971-1980	4662 (13.6)	569 (11.4)	
1961-1970	3846 (11.2)	353 (7.0)	
< 1961	1865 (5.4)	154 (3.1)	< 0.0001
Age (years) at first ADHD diagnosis, *median*, *range*	18 (3-80)	16 (3-80)	< 0.0001
Age (years) at first ASD diagnosis, *median*, *range*		16 (3-80)	
**Age (years) at first ADHD diagnosis**			
3-12	9143 (26.6)	1699 (33.9)	
13-17	7784 (22.7)	1070 (21.4)	
18-29	8120 (23.6)	1325 (26.4)	
30-39	4515 (13.1)	502 (10.0)	
40-49	3460 (10.1)	326 (6.5)	
50-59	1171 (3.4)	78 (1.6)	
> 60	181 (0.53)	12 (0.24)	< 0.0001
**Age (years) at first ASD diagnosis,**			
3-12		1708 (34.1)	
13-17		1130 (22.6)	
18-29		1269 (25.3)	
30-39		485 (9.7)	
40-49		323 (6.4)	
50-59		86 (1.7)	
> 60		11 (0.22)	
**ASD diagnosed** ***before*** **ADHD**		1267 (25.3)	
**ASD and ADHD diagnosed on the same date**		2167 (43.2)	
**ASD diagnosed** ***after*** **ADHD**		1578 (31.5)	
**Comorbid diagnoses diagnosed before ADHD**			
Depression	6580 (19.1)	944 (18.8)	0.60
Anxiety disorders (excluding OCD)	6397 (18.6)	894 (17.8)	0.19
SUD Alcohol	4062 (11.8)	367 (7.3)	< 0.0001
SUD Narcotics	3879 (11.3)	323 (6.4)	< 0.0001
Suicidal attempt	3500 (10.2)	445 (8.9)	0.0041
Stress related disorders and PTSD	3249 (9.5)	397 (7.9)	0.0005
Bipolar disorder	1782 (5.2)	199 (4.0)	0.0002
Intellectual disability	1224 (3.6)	399 (8.0)	< 0.0001
Psychotic disorder	868 (2.5)	180 (3.6)	< 0.0001
OCD	773 (2.3)	260 (5.2)	< 0.0001
Eating disorder	701 (2.0)	113 (2.3)	0.32
Schizophrenia	237 (0.7)	55 (1.1)	0.0017

*ADHD* attention -deficit /hyperactivity disorder, *ASD* autism spectrum disorder, *OCD* obsessive-compulsive disorder, *SUD* substance use disorder, *PTSD* post-traumatic stress disorder

*P values from Pearson *χ*2 tests or Wilcoxon rank-sum test

From the cohort of individuals with ADHD, we identified those with two or more diagnoses of ASD appearing after age three, according to ICD-9 (299) and ICD-10 (F84.1, F84.5, F84.8, F84.9), but the first ASD diagnosis had to appear before January 1, 2011. ADHD and ASD are both neurodevelopmental disorders, and to be correctly diagnosed symptom onset is required from childhood. Therefore, the first ASD date that appears in the register is not an indicator of onset, but instead indicates when the diagnosis first was decided on in conjunction with the neuropsychiatric assessment. Subsequently, we considered individuals as ASD cases regardless if the first ASD date appeared before or after the first ADHD date (Supplement).

### Prescriptions of ADHD medication in Sweden

In Sweden, ADHD medications are usually prescribed in specialized psychiatric units, but may also be prescribed by medical doctors specialized in, e.g., pediatric neurology. Individuals must have a documented neuropsychiatric assessment that resulted in an ADHD diagnosis. Between the years of 2005 and 2013, the substances methylphenidate and atomoxetine were approved drugs for ADHD treatment in Sweden by the Swedish Medical Products Agency and recommended for first-line treatment (Swedish Medical Agency). Prescription of dexamphetamine, and amphetamine substances (e.g., Adderall) required a special application to the Swedish Medical Products Agency during the study period. Modafinil was not approved for ADHD treatment, but was prescribed off-label for ADHD. Short-acting and extended release formulations were dispensed for methylphenidate and amphetamines, while for dexamphetamine only short acting formulations were dispensed.

### ATC codes for ADHD medication

From the PDR, we identified filled prescriptions for the following substances according to the ATC drug class: amphetamine (N06BA01), dexamphetamine (N06BA02), methylphenidate (N06BA04), modafinil (N06BA07), and atomoxetine (N06BA09). There were no dispensations recorded for lisdexamfetamine (N06BA12), guanfacine (C02AC02), dextromethamphetamine (N06BA03), and dexmethylphenidate (N06BA11) during the study period and these were not included in the analysis.

### Definitions for treatment with ADHD medication

We defined treatment periods for ADHD medication overall as “no treatment” (no filled prescription from any ADHD medication), “single dispense” (one filled prescription from any ADHD medication) and “continuous treatment” (a sequence of two filled prescriptions from one ADHD medication at separate dates where the gap did not exceed 90 days).

Treatment periods were defined for each separate ADHD medication (i.e., methylphenidate, atomoxetine, dexamphetamine, modafinil, and amphetamine) as “no treatment” or “continuous treatment” (See Supplement for details). Each individual was followed during three years, starting from the first period of “continuous treatment”, and it was possible for one single individual to start a “continuous treatment” with several types of ADHD-medications, either sequentially or simultaneously, and was therefore included in more than one of the separate ADHD medications analyses.

### Definitions for methylphenidate daily doses

We studied an approximation of doses per day for methylphenidate based on filled prescriptions in individuals who started a continuous treatment with methylphenidate between July 1, 2005, and January 1, 2011. Individuals were followed during a 3-year period and information on dose and number of items per filled prescription was used to calculate the total dose per 6 months (Supplement Figure 2). The dose was divided with number of days to receive an approximation of milligram per day (mg/day). The total dose was set to missing if there were no dispensations during a 6-month interval.

### Covariates

We adjusted for sex, birth year categories, year of first ADHD diagnosis (Supplement Table 1), and psychiatric comorbidities. The comorbid diagnosis had to appear before the first ADHD diagnosis. The following comorbid diagnoses were adjusted for substance use disorder (alcohol or narcotics), depression, anxiety disorder, obsessive compulsive disorder (OCD), any suicidal attempt, stress-related disorders and post-traumatic stress disorder (PTSD), bipolar disorder, psychotic disorder, schizophrenia, eating disorder, and intellectual disability, for ICD-codes see supplement methods.

### Statistical analysis

Descriptive statistics were presented as frequencies, percentages, or medians with minimum and maximum values. Group differences were calculated with Pearson *χ*2 tests for categorical data or Wilcoxon rank-sum test for continuous data.

First, we examined whether individuals with ADHD and coexisting ASD are less likely to initiate treatment with ADHD medications overall in all individuals. We then separated into age-categories based on age when the first ADHD diagnosis appeared in the register (adults, age ≥ 18 years, adolescents, age 13-17 years, and children, age ≤ 12 years). We fitted logistic regression models (SAS PROC LOGISTIC) and analyzed the odds for continuous treatment versus no treatment, which included those who had a single dispense. In a secondary analysis, we analyzed the odds for continuous treatment versus no treatment and continuous treatment versus one single dispense. Odds ratios (ORs) were calculated with the associated two-sided 95% confidence intervals (CIs), for the likelihood of being prescribed ADHD medication, comparing individuals with ADHD without coexisting ASD to individuals with ADHD with coexisting ASD. First, we fitted a model without any other covariates, except for sex and birth categories (Birth year< 1961, 1961-1970, 1971-1980, 1981-1990, 1991-2000, > 2000). Next, we included covariates adjusting for year of first ADHD diagnosis as a continuous linear covariate and the presence of psychiatric comorbidities (yes/no), which could be suspected to confound our OR estimates.

Second, in those who started a continuous treatment, we examined whether individuals with ADHD and coexisting ASD are more likely to be prescribed second line treatment options (i.e., dexamphetamine, amphetamine, or modafinil). We fitted a logistic regression model for each type of ADHD medication in all individuals and separated into age-categories defined as in the first analysis.

Third, we examined whether individuals with ADHD and coexisting ASD were dispensed different average doses of ADHD medication. Due to non-normal distribution of the data, we applied the Wilcoxon rank-sum test (SAS PROC NPAR1WAY). Methylphenidate doses per day (mg/day) were calculated over six-month intervals, during a total time-period of three years. We also stratified into age-categories defined as in the first analysis. The Wilcoxon rank sum test is a non-parametric test that does not require the data to follow a particular data distribution, e.g., normal distribution, and is robust against single gross outliers [21]. Two-sided*p* values of < 0.05 corresponding to two-sided 95% confidence intervals not covering the value one were considered as statistically significant. 

Sensitivity analyses were performed, by excluding individuals with their first ADHD diagnosis at age 40 or later, to ascertain that the results were not biased by possible differences regarding coexisting ADHD and ASD in young adults as compared to elderly. All statistical analyses were performed using SAS 9.4.

## Results

We identified 39,386 individuals with a diagnosis of ADHD, of which 5012 (13%) had a coexisting diagnosis of ASD. The median age at first ADHD diagnosis was lower in individuals with ADHD and coexisting ASD (ADHD, 18 years [range 3-80]; ADHD with ASD, 16 years [range 3-80]), and the median age at first ASD diagnosis was 16 years (range 3-80, Table 1). In individuals with a diagnosis of ASD, 43% had their first diagnosis on the same date as their first ADHD diagnosis, 25% had the ASD diagnosis before, and 32% after the first ADHD diagnosis (Table 1). Intellectual disability, psychotic disorder, OCD, and schizophrenia diagnoses were more common as comorbid diagnoses in individuals with ADHD and coexisting ASD, while substance use disorder (alcohol or narcotics), suicidal attempt, stress-related disorders including PTSD, and bipolar disorder were more common in individuals with ADHD without ASD (Table 1).

### Treatment with any ADHD medication

Individuals with ADHD and coexisting ASD were less likely to start continuous treatment with ADHD medication after adjusting for sex, age, year of first ADHD diagnosis and psychiatric comorbidities (OR, 0.80; 95% CI, 0.75-0.86; Table 2). When analyzed separately in adults, adolescents and children similar results were observed (Table 2). In a secondary analysis, continuous treatment was compared with those who had received one single dispense or no dispense at all. Individuals with coexisting ADHD and ASD continued to be less likely to receive treatment when compared to those who received no dispenses at all, while the comparison to those receiving one dispense did not reach a statistically significant level (Supplement Table 2). Due to the wide age range in the sample, we performed sensitivity analyses in which individuals who received their first ADHD diagnosis at age 40 or later on were excluded. There were no changes in main results regarding start of continuous treatment with ADHD medication (Supplement Table 3).

**Table 2:** Likelihood for starting continuous treatment with medication for attention-deficit/hyperactivity disorder (ADHD) comparing individuals with and without autism spectrum disorder (ASD)

	ADHD without coexisting ASD	ADHD with coexisting ASD	Model 1^**#**^	Model 2^**##**^
	Frequency (%)	Frequency (%)	Odds ratio (95% confidence interval)	Odds ratio (95% confidence interval)
**All individuals**	*n* = 34,374	*n* = 5012		
No treatment	6706 (19.5)	1191 (23.8)		
Continuous treatment	27,668 (80.5)	3821 (76.2)	0.77 (0.72-0.83)^###^	0.80 (0.75-0.86)^###^
**Adults**	*n* = 17447	*n* = 2243		
No treatment	4062 (23.3)	621 (27.7)		
Continuous treatment	13,385 (76.7)	1622 (72.3)	0.82 (0.74-0.91)^###^	0.85 (0.77-0.94)^###^
**Adolescents**	*n* = 7784	*n* = 1070		
No treatment	1319 (17.95)	237 (22.15)		
Continuous treatment	6465 (83.05)	833 (77.85)	0.74 (0.63-0.86)^###^	0.76 (0.65-0.89)^###^
**Children**	*n* = 9143	*n* = 1699		
No treatment	1325 (14.5)	333 (19.6)		
Continuous treatment	7818 (85.5)	1366 (80.4)	0.72 (0.63-0.82)^###^	0.76 (0.66-0.87)^###^

Note: An odds ratio below one indicate that individuals with ADHD and ASD are less likely to receive medication. Included medications in the analysis: methylphenidate, atomoxetine, dexamphetamine, modafinil, and amphetamine. “No treatment”, individuals with zero or one single filled prescription. “Continuous treatment”. two or more filled prescriptions. Age categories based on age at first ADHD diagnosis (adults, age ≥ 18 years; adolescents, age 13-17 years; children, age ≤ 12 years)

^**#**^Adjusted for sex and birth categories in years, and year of first ADHD diagnosis

^**##**^Adjusted for sex, birth categories in years, year of first ADHD diagnosis, and psychiatric comorbidities before first dispense date

^###^*p* value < 0.0001

### Treatment with different types of ADHD medications

Among individuals who started a continuous treatment with ADHD medication, we analyzed the likelihood for starting a continuous treatment for different types of ADHD medications. Methylphenidate was the most commonly dispensed medicine, followed by atomoxetine (Table 3). Individuals with ADHD and coexisting ASD were less likely to start continuous treatment with methylphenidate (OR, 0.86; 95% CI, 0.77-0.97), while no statistically significant difference was seen for atomoxetine (OR, 1.1; 95% CI, 0.98-1.2; Table 3). Individuals with ADHD and coexisting ASD were more likely to start continuous treatment with any of the second line treatment choices dexamphetamine (OR, 1.7; 95% CI, 1.3-2.1), modafinil (OR, 1.6; 95% CI, 1.2-2.2), and amphetamine (OR, 1.8; 95% CI, 1.3-2.6; Table 3). Similar results were observed before and after adjusting for year of first ADHD diagnosis and psychiatric comorbidities.

**Table 3:** Among individuals who started continuous treatment with medication for attention-deficit/hyperactivity disorder (ADHD): Likelihood for receiving a specific drug treatment comparing individuals with ADHD and coexisting autism spectrum disorder (ASD) to individuals with ADHD without coexisting ASD

	ADHD without coexisting ASD	ADHD with coexisting ASD	Model 1^a^	Model 2^b^
	Frequency (%)	Frequency (%)	Odds ratio (95% confidence interval)	Odds ratio (95% confidence interval)
**All individuals**	***n*** **= 27,668**	***n*** **= 3821**		
Methylphenidate	25,508 (92.2)	3467 (90.8)	**0.84 (0.74-0.94)**	**0.86 (0.77-0.97)**
Atomoxetine	6553 (23.7)	1003 (26.3)	1.07 (0.99-1.16)	1.06 (0.98-1.15)
Dexamphetamine	457 (1.7)	87 (2.3)	**1.6 (1.3-2.1)**	**1.7 (1.3-2.1)**
Modafinil	303 (1.1)	60 (1.6)	**1.7 (1.2-2.2)**	**1.6 (1.2-2.2)**
Amphetamine	142 (0.51)	39 (1.0)	**1.9 (1.3-2.7)**	**1.8 (1.3-2.6)**
**Adults**	***n*** **= 13,305**	***n*** **= 1622**		
Methylphenidate	12327 (92.1)	1477 (91.06)	0.89 (0.74**-**1.1)	0.92 (0.77**-**1.1)
Atomoxetine	2577 (19.25)	317 (19.54)	0.98 (0.86**-**1.1)	0.97 (0.85**-**1.1)
Dexamphetamine	413 (3.09)	82 (5.06)	**1.8 (1.4-2.3)**	**1.9 (1.5-2.4)**
Modafinil	295 (2.2)	55 (3.39)	**2.1 (1.3-3.4)**	**2.2 (1.3-3.5)**
Amphetamine	80 (0.6)	22 (1.36)	**1.6 (1.2-2.1)**	**1.5 (1.1-2.1)**
**Adolescents**	***n*** **= 6465**	***n*** **= 833**		
Methylphenidate	5922 (91.6)	757 (90.9)	0.93 (0.72**-**1.2)	0.99 (0.76-1.3)
Atomoxetine	1692 (26.2)	229 (27.5)	1.0 (0.88**-**1.2)	1.1 (0.87-1.2)
Dexamphetamine	21 (0.32)	3 (0.36)	0.91 (0.27**-**3.1)	0.71 (0.2-2.5)
Modafinil	6 (0.09)	3 (0.36)	3.9 (0.96**-**15.9)	3.5 (0.78-15.4)
Amphetamine	36 (0.56)	7 (0.84)	1.4 (0.6**-**3.06)	1.3 (0.55-2.9)
**Children**	***n*** **= 7818**	***n*** **= 1366**		
Methylphenidate	7259 (92.9)	1233 (90.3)	**0.72 (0.59-0.88)**	**0.73 (0.60-0.90)**
Atomoxetine	2284 (29.2)	457 (33.5)	**1.1 (1.0-1.3)**	**1.1 (1.0-1.3)**
Dexamphetamine	23 (0.29)	2 (0.15)	0.41 (0.10**-**1.7)	0.40 (0.09-1.7)
Modafinil	2 (0.03)	2 (0.15)	6.02 (0.85**-**42.8)	5.47 (0.7739.1)
Amphetamine	26 (0.33)	10 (0.73)	**2.23 (1.07-4.6)**	1.77 (0.83-3.8)

Note: An odds ratio above one indicate that individuals with ADHD and coexisting ASD are more likely to receive medication and an odds ratio below one indicate that those individuals are less likely to receive medication. Significant odds ratios are marked as bolded

Dexamphetamine and amphetamine required a separate application to the Swedish Medical Agency during the observation period (2005-2013). Age categories based on age at first ADHD diagnosis (adults, age ≥ 18 years, adolescents, age 13-17 years, children, age ≤ 12 years)

^a^Adjusted for sex and birth categories in years and year of first ADHD diagnosis

^b^Adjusted for sex, birth categories in years, year of first ADHD diagnosis, and psychiatric comorbidities before first dispense date

We then stratified the analysis into adults, adolescents, and children. Adults with ADHD and ASD were more likely to start treatment with dexamphetamine, modafinil, or amphetamine. Children diagnosed with ADHD and coexisting ASD were less likely to initiate treatment with methylphenidate but were more likely to initiate treatment with atomoxetine (Table 3). No significant differences were seen in adolescents. Also here, we performed an analysis in which adults diagnosed with ADHD at age 40 or above were excluded, but no changes from the main results were observed (Supplement Table 4).

### Methylphenidate treatment during a three-year observation period

**Fig. 1:**
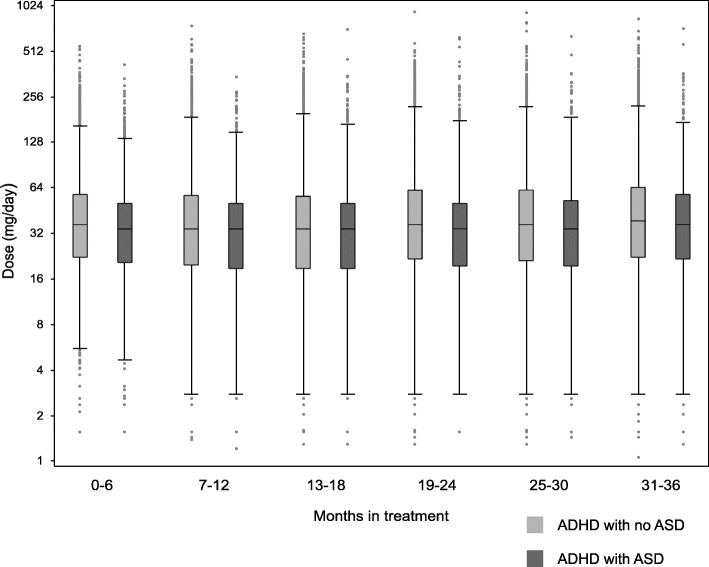
Methylphenidate doses milligram per day (mg/day) in individuals with attention-deficit/hyperactivity disorder (ADHD) with or without co-existing autism spectrum disorder (ASD). Side by side, box plots where each box is drawn between the 25th and 75th percentile representing the central 50% of the sample. Individual values (dots) for the 1% highest and lowest doses

We studied doses of methylphenidate during a three-year observation period. By the end of the three years, 61% of the individuals with ADHD and 63% of the individuals with ADHD and ASD continued to receive dispensations from methylphenidate. Individuals with ADHD and coexisting ASD were prescribed statistically significantly lower doses of methylphenidate (Table 4, Fig. 1). The differences in median doses over the three years were not exceeding 4 mg/day. When we stratified the analysis into adults, adolescents, and children, the differences in dose per day were significant during the three-year period in the adult population (Table 4). Notably, we observed considerable variation in dispensed daily doses, and the highest dose for methylphenidate was 1065 mg/day over a six-month period, which is an exceptionally high daily dose as compared to clinical guidelines that recommends daily doses up to around 100 mg/day for methylphenidate [22]. We performed an additional analysis in which the 1% highest and lowest doses were removed which did not change the results considerable (Supplement Table 5).

**Table 4:** Attention-deficit/hyperactivity disorder (ADHD) medication mean doses (mg/day) in 6 months intervals, comparing individuals with ADHD with coexisting autism spectrum disorder (ASD) versus ADHD without coexisting ASD, and stratified analyses by sex, age category (adults/adolescents/children), substance use disorder (SUD), and intellectual disability (ID)

	ADHD without coexisting ASD			ADHD with coexisting ASD				
	Number of subjects (%)	Median	Range doses (mg)	Number of subjects	Median	Range doses (mg)	***Z*** value	***p*** value
**All, months**								
**0-6**	25508 (100)	39.9	2-570	3467 (100)	35.7	2-437	-8.97	< 0.0001
**7-12**	20661 (81)	35.5	1-799	2759 (80)	35.5	1-368	-6.32	< 0.0001
**13-18**	18420 (72)	35.5	1-1065	2505 (72)	35.5	1-762	-4.56	< 0.0001
**19-24**	17471 (68)	39.4	0.01-1058	2373 (68)	35.5	2-669	-4.46	< 0.0001
**25-30**	16393 (64)	39.4	1-943	2269 (65)	35.5	1-674	-4.70	< 0.0001
**31-36**	15508 (61)	41.4	1-879	2171 (63)	38.4	1-762	-3.55	0.0004
**Adults, months**								
**0-6**	12327 (100)	50.3	2-570	1477 (100)	42.9	2-437	-5.35	< 0.0001
**7-12**	9661 (78)	52.6	1-799	1129 (76)	44.4	2-368	-3.94	< 0.0001
**13-18**	8433 (68)	52.6	2-1065	1020 (69)	44.4	1-762	-4.04	< 0.0001
**19-24**	7960 (65)	53.2	0.01-1058	936 (63)	47.3	2-669	-2.49	0.0047
**25-30**	7435 (60)	53.2	1-943	894 (61)	46.4	1-674	-3.85	< 0.0001
**31-36**	7032 (57)	53.2	1-879	840 (57)	53.2	1-762	-2.87	0.0016
**Adolescents, months**								
**0-6**	5922 (100)	38.4	3-273	757 (100)	38.4	3-142	-0.55	0.37
**7-12**	4597 (78)	35.5	2-218	597 (79)	35.5	3-124	1.83	0.58
**13-18**	3958 (67)	32.5	1-256	516 (68)	35.5	2-177	1.96	0.067
**19-24**	3648 (62)	35.5	1-251	477 (63)	35.5	2-191	2.13	0.051
**25-30**	3273 (55)	35.5	1-253	446 (59)	35.5	2-160	2.20	0.033
**31-36**	2965 (50)	35.5	1-266	427 (56)	35.5	1-181	-0.89	0.028
**Children, months**								
**0-6**	7259 (100)	31.2	2-182	1233 (100)	28.3	3-112	-5.35	< 0.0001
**7-12**	6403 (88)	29.6	2-195	1033 (84)	29.6	1-127	-3.94	0.0021
**13-18**	6029 (83)	29.6	1-222	969 (79)	29.6	1-104	-4.04	0.099
**19-24**	5863 (801)	35	1-222	960 (78)	31.5	2-149	-2.49	0.0002
**25-30**	5685 (78)	35.5	1-163	929 (75)	32.9	2-160	-3.85	0.0030
**31-36**	5511 (76)	35.5	1-305	904 (73)	35.5	2-210	-2.87	0.062

*ADHD* attention deficit hyperactivity disorder, *ASD* autism spectrum disorder, *mg* milligram

^#^Wilcoxon rank sum test used for group comparisons of doses

A negative *Z* value and a *p* value below 0.05 indicate that individuals with ADHD and coexisting ASD are prescribed statistically significantly lower doses as compared to individuals without coexisting ASD

Age categories based on age at first ADHD diagnosis (adults, age ≥ 18 years, adolescents, age 13-17 years, children, age ≤ 12 years)

## Discussion

This population-based study with prospective follow-up on 39,386 individuals with ADHD with or without coexisting ASD found that individuals with coexisting ASD were at lower odds to start continuous treatment with ADHD medication and this was observed in adults, adolescents as well as in children. In those who had started treatment, children with ADHD and coexisting ASD were less likely to receive prescriptions from methylphenidate and more likely to receive prescriptions from atomoxetine, while adults with coexisting ASD were more likely to receive second line treatment choices, i.e., dexamphetamine, amphetamine, or modafinil. Furthermore, adults with coexisting ASD were prescribed, on average, lower doses of methylphenidate over a period of three years.

Evidence for medical treatment of ADHD in ASD is limited and to our knowledge, there are no previous pharmacoepidemiological studies directly comparing dispenses and doses of ADHD medications in ADHD with or without coexisting ASD. However, most studies within the field conclude that ADHD medications, such as methylphenidate and atomoxetine, have effect on ADHD symptoms in ASD [15, 16, 19, 23–26]. Also, clinical guidelines for ADHD treatment recommend that individuals with ADHD and coexisting ASD should be offered the same medication choices as those with ADHD only [6]. Our data show that 81% of the individuals with ADHD without ASD started continuous treatment, while in ASD, 76% started with ADHD medication. Overall, the proportions of treated individuals were in line with a previous Swedish study on cross-sectional data from the PDR [27]. On the other hand, the number of treated individuals was higher in comparison to results from a Danish register study [28], and lower in relation to a US cohort in which 86% of children with combined ASD and ADHD received psychostimulants [29]. Yet, our findings indicate that individuals with ADHD and coexisting ASD and regardless of age group are less likely to start continuous treatment with ADHD medication.

For the different medication types, we found that adults with ADHD and ASD were more likely to initiate treatments with dexamphetamine, modafinil, or amphetamine, and the results did not change after adjusting for a number of comorbidities including substance use disorder. In Sweden, during the period covered, ADHD patients were obliged to try out methylphenidate and/or atomoxetine with unacceptable side effects, or a marked insufficient effect, before being prescribed dexamphetamine or amphetamine. Thus, our findings lead us to speculate whether individuals with ASD may switch from methylphenidate to second line alternatives due to a worse response, or side effects from ADHD medication, as previously reported [13, 16, 17]. We also found that children with ADHD and coexisting ASD were more likely to be prescribed atomoxetine and less likely to be prescribed methylphenidate. Data on treatment effects from atomoxetine in children with coexisting ADHD and ASD is scarce, although effects on hyperactivity has been demonstrated [23, 30, 31]. Whether this is due to preferences of the prescriber, or due to a worse treatment response to methylphenidate as compared to children without ASD [32], remains unclear. It seems, however, unlikely that the higher prescription rate of atomoxetine in children with ADHD and coexisting ASD is due to requests from the child’s guardian.

Individuals with ADHD and coexisting ASD were on average prescribed lower methylphenidate doses and this difference was most pronounced in adults. The use of lower methylphenidate doses in ASD may be an indicator of differences in tolerance, treatment response, or that lower doses are sufficient when there is a treatment effect. One previous study found that individuals with ADHD and previous substance use disorder were prescribed 40% higher methylphenidate doses as compared to individuals with ADHD without substance use disorder [33]. The finding was interpreted as a need for higher methylphenidate doses in individuals with ADHD and coexisting substance use disorder, although the role of the patients’ demands for higher doses were not explored. The differences found in the present study between ADHD with and without ASD were considerably smaller, and it is not certain that the identified differences have a clinical significance.

The median age for the first ADHD and ASD diagnosis was relatively high, 18 years for individuals with ADHD and 16 years for individuals with ADHD and ASD. These results are however in agreement with previous studies using Swedish register data [27] and is most likely an effect of increased diagnostics in adults being diagnosed with ADHD later in life.

### Limitations

The findings of the study must be interpreted in the light of several limitations. The data reflect filled prescriptions medications, but we do not have information on medications prescribed but were never dispensed, or whether the medications were consumed. Previous studies found that medical adherence in ADHD is low and may vary between 13 and 24% [34]. However, our requirements of at least two subsequent filled prescriptions may partly compensate for this. The analyses of average doses are approximations based on filled prescriptions, with no access to the written instruction from the prescriber, and with no information on whether individuals were receiving medications from several prescribers at the same time. The wide range of the estimated average daily doses probably reflects these limitations as some of the extreme values of calculated daily doses are not clinically relevant. On the other hand, this limitation would not differ between the studied groups. More importantly, we did not have information on further phenotypic characteristics, and here bodyweight is the most important factor as it affect doses of methylphenidate prescribed [35]. Further, we did not study other simultaneous pharmacological treatments, which may affect doses and type of ADHD medications prescribed. For example, other types of psychotropic medications may potentially differ between the groups. Here our strategy to adjust for psychiatric comorbidities would imply that such an effect is likely not to confound the present results. However, as we took into account comorbidities preceding the ADHD diagnosis there is still a possibility that comorbid conditions diagnosed afterwards would have an impact of results. Finally, individuals with undiscovered comorbidity of ADHD and ASD could be misclassified from the register data, which may possibly result in reduced differences between the groups studied.

### Clinical and research implications

The study identified differences in the treatment with ADHD medication between adults, and to some extent adolescents  and children, with or without coexisting ADHD and ASD regarding treatment initiation, medication type, which could not be explained by other comorbid psychiatric disorders. Given that the prescribing patterns differed in individuals with coexisting ASD, the clinician needs to be aware not only of possible variances in response patterns or side effects from medications but also of differences regarding the individuals’ preferred choices of pharmacological treatment. The findings do not support a different treatment strategy regarding ADHD medication in individuals with coexisting ASD, but this patient population should be subject to more careful monitoring and dose-titration, and may be in greater need for clinical interventions and support other than pharmacological treatment. Further clinical studies are needed, particularly longitudinal studies, addressing potential differences in prescribing patterns, and pharmacological effects from ADHD medications that directly compares individuals with ADHD with and without coexisting ASD. Although the study is restricted to individuals in Sweden, the clinical diagnoses and the studied medications are now standard across the western world [8] and we believe the results would be predictive also for other populations.

## Conclusion

This is the first study to investigate the prescribing patterns of ADHD medication in ADHD with and without coexisting ASD. The findings from this study indicate differences between individuals with and without ASD. If these are due to clinicians’ perceptions of medication effects in patients with ASD, or due to different medication responses in patients with ASD, need to be addressed in future studies. Meanwhile, patients with ASD should be subject to careful monitoring and dose-titration in the clinic.

## Supplementary Information

**Additional file 1.**

## Data Availability

The datasets generated and/or analyzed during the current study are not publicly available due to legal reasons but may be available from the corresponding author on reasonable request.

## Abbreviations

ADHD: Attention-deficit/hyperactivity disorder
RCT: Randomized clinical trial
ASD: Autism spectrum disorder
NPR: National Patient Register
ICD: International classification of diseases
PDR: Prescribed Drug Register
ATC: Anatomic Therapeutic Chemical classification system
OCD: Obsessive compulsive disorder
PTSD: Post-traumatic stress disorder
OR: Odds ratios (ORs)
CI: Confidence intervals
