# Supplementary material for: Medications for attention-deficit/hyperactivity disorder in individuals with or without coexisting autism spectrum disorder: analysis of data from the Swedish prescribed drug register
Source: J Neurodev Disord. 2020 Dec 23;12:44. doi: 10.1186/s11689-020-09352-z (PMC7758935; doi:10.1186/s11689-020-09352-z)
Supplement: Supplementary file 1 — Additional file 1. [file 11689_2020_9352_MOESM1_ESM.docx]

**Supplement**

Johansson et. al. Medication in individuals with attention-deficit/hyperactivity disorder and coexisting autism spectrum disorder: analysis of data from the Swedish Prescribed Drug register

**Supplement - methods**

**Supplement table 1**

**Supplement table 2**

**Supplement table 3**

**Supplement table 4**

**Supplement table 5**

**Supplement figure 1**

**Supplement figure 2**

**Supplement references**

**Supplement - methods**

***Description of health care registers in Sweden***

To obtain register linkages between the different national registers we used the national personal identification number assigned to each resident in Sweden.^1^ The following health care registers were used:

- The National Patient Register in Sweden (NPR, National Board of Health and Welfare) contains information on all hospitalizations for psychiatric diagnoses from 1973, and diagnoses from specialist outpatient care from 2001. The register diagnosis of NPR was previously validated.^2^
- Total Population Register (Statistics Sweden), which includes information on all residents in Sweden.^3^
- The Swedish Prescribed Drug Register contains data about all filled prescriptions from pharmacies in Sweden from July 1, 2005 and onwards with information on brand, drug class according to the Anatomical Therapeutic Chemical [ATC] Classification System, number of items (e.g. tablets or capsules), doses, and dispensation dates.^4^

***Diagnostic codes used in the study***

All diagnostic codes were identified from NPR, according to the International classification of diseases (ICD). Note that NPR includes all psychiatric hospitalizations from 1973 and diagnoses from specialized out-patient care from 2001.

The following diagnostic codes were used:

ADHD (ICD-10 F90), Autism spectrum disorder (ICD-9: 299, ICD-10: F84.1-F84.5, F84.8-F84.9, Substance use disorder, alcohol abuse (ICD-9 303, 305A; ICD-10 F10), Substance use disorder, drug abuse (ICD-9 304, 305X; ICD-10 F11-F16, F18, F19), Schizophrenia (ICD-9 295; ICD-10 F20), Psychosis, other (ICD-9 297, 298; ICD-10 F21-F29), Bipolar disorder (ICD-9 296 [excluding 296B]; ICD-10 F30, F31), Depression (ICD-9 296B, 300E, 311; ICD-10 F32, F33), Anxiety disorders (ICD-9 300A, 300C; ICD-10 F40, F41) Obsessive compulsive disorder (ICD-9 300D; ICD-10 F42), Stress related disorders and PTSD (ICD-9 308, 309A, 309B, 309W, 309X; ICD-10 F43), Eating disorders (ICD-9 307B, 307F; ICD-10 F50), Intellectual disability (ICD-9 317-319; ICD-10 F70-F73, F78, F79), Suicide (ICD-9 E95A-E95H, E95W [or E950-E959]; ICD-10 X60-X84).

For the diagnoses of ASD it is important to note that diagnostic procedures in clinical practice in Sweden was during the study period based on diagnoses from ICD-10 and the Diagnostic Manual of Mental disorders (DSM-IV). According to DSM-IV it was not possible to combine the diagnosis of ADHD and ASD, but in clinical practice this criteria was not strictly followed. Therefore, combined diagnoses of ADHD and ASD exist in the register, but the prevalence of ASD in individuals with ADHD may have been underestimated due to the DSM-IV criteria. This may result in an underestimation of the prevalence in combined ADHD and ASD diagnosis as commented as a limitation in the discussion section of the manuscript.

**Supplement table 1**. Distribution of when the first ADHD diagnosis appears in the national patient register (NPR) from 2005 through 2010 in the sample.

| **Year of first diagnosis** | **N** | **%** |
| --- | --- | --- |
| 2005 | 1224 | 3.11 |
| 2006 | 4033 | 10.24 |
| 2007 | 5896 | 14.97 |
| 2008 | 7803 | 19.81 |
| 2009 | 9751 | 24.76 |
| 2010 | 10679 | 27.11 |
| **Total** | **39386** | **100** |

**Supplement table 2.** Likelihood of starting treatment with medication for attention deficit hyperactivity disorder (ADHD) in individuals with and without autism spectrum disorder (ASD). Comparing continuous treatment ( ≥2 filled prescriptions), one filled prescription, and no treatment.

|  | **ADHD without co-existing ASD** | **ADHD with co-existing ASD** | **Model 1^#^** | **Model 2^##^** |
| --- | --- | --- | --- | --- |
|  | **Frequency** | **Frequency** | **Odds-ratio**  **(95% confidence interval)** | **Odds-ratio**  **(95% confidence interval)** |
| **Continuous treatment vs. No treatment** | 27668 vs. 3937 | 3821 vs. 735 | 0.73  (0.67-0.80) ^###^ | 0.77  (0.70-0.84) ^###^ |
| **Continuous treatment vs. One filled prescription** | 27668 vs. 2769 | 3821 vs. 456 | 0.87  (0.77-0.99) ^###^ | 0.88  (0.77-1.0) |

Note: An odds-ratio below one indicate that individuals with ADHD and ASD are less likely to receive medication. Included medications in the analysis: Methylphenidate, atomoxetine, dexamphetamine, modafinil and amphetamine. “No treatment”: Individuals with zero filled prescriptions. “One filled prescription”: Individuals with one single filled prescription. “Continuous treatment”: Two or more filled prescriptions.

**^#^**Adjusted for sex and birth categories in years and year of first ADHD diagnosis. **^##^**Adjusted for sex, birth categories in years, year of first ADHD diagnosis, and psychiatric comorbidities before first dispense date. ^###^ P-value < 0.001.

**Supplement table 3**. Sensitivity analysis excluding individuals who received their first ADHD-diagnosis at age 40 or later. Likelihood for starting continuous treatment with any medication for attention-deficit/hyperactivity disorder (ADHD) comparing individuals with and without autism spectrum disorder (ASD).

|  | **Model 1^#^** |  | **Model 2^##^** |  |
| --- | --- | --- | --- | --- |
|  | **Odds-ratio**  **(95% confidence interval)** | ***p*-value** | **Odds-ratio**  **(95% confidence interval)** | ***p*-value** |
|  | **n = 29562** |  | **n = 4596** |  |
| **All individuals**  **(< 40 years)** | 0.77 (0.72 - 0.83) | < 0.0001 | 0.81 (0.75 - 0.87) | < 0.0001 |
|  | **n = 12635** |  | **n = 1827** |  |
| **Adults**  **(18 to 39 years)** | 0.83 (0.75 - 0.93) | 0.0014 | 0.88 (0.78 - 0.98) | 0.021 |

**^#^**Adjusted for sex and birth categories in years and year of first ADHD diagnosis. **^##^**Adjusted for sex, birth categories in years, year of first ADHD diagnosis, and psychiatric comorbidities before first dispense date.

**Supplement table 4.** Sensitivity analysis excluding individuals who received their first ADHD-diagnosis at age 40 or later. Likelihood for starting continuous treatment with medications for attention-deficit/hyperactivity disorder (ADHD). Comparing individuals with ADHD and coexisting autism spectrum disorder (ASD) to individuals with ADHD without coexisting ASD.

|  | **ADHD without coexisting ASD** | **ADHD with coexisting ASD** | **Model 1^#^** | **Model 2^##^** |
| --- | --- | --- | --- | --- |
|  | **Frequency (%)** | **Frequency (%)** | **Odds-ratio**  **(95% confidence interval)** | **Odds-ratio**  **(95% confidence interval)** |
| **All individuals (3 to 39 years at first ADHD diagnosis)** | **n = 23944** | **n= 3520** |  |  |
| Methylphenidate | 22082 (92.2) | 3200 (90.9) | **0.85 (0.75-0.97)** | 0.89 (0.78-1.0) |
| Atomoxetine | 5879 (24.6) | 952 (27.1) | 1.1 (1.0-1.2) | 1.1 (0.98-1.2) |
| Dexamphetamine | 287 (1.2) | 66 (1.9) | **1.6 (1.3-2.2)** | **1.7 (1.3-2.2)** |
| Modafinil | 203 (0.85) | 48 (1.4) | **1.7 (1.3-2.4)** | **1.7 (1.2-2.4)** |
| Amphetamine | 113 (0.47) | 34 (0.97) | **1.9 (1.3-2.8)** | **1.8 (1.2-2.7)** |
| **Adults (18 to 39 years at first ADHD diagnosis)** | **n=9,661** | **n=1,321** |  |  |
| Methylphenidate | 8901 (92.1) | 1210 (91.6) | 0.96 (0.78-1.2) | 1.0 (0.81-1.2) |
| Atomoxetine | 1903 (19.7) | 266 (20.1) | 1.0 (0.86-1.2) | 0.99 (0.85-1.1) |
| Dexamphetamine | 243 (2.5) | 61 (4.6) | **1.9 (1.4-2.5)** | **1.9 (1.4-2.6)** |
| Modafinil | 195 (2.0) | 43 (3.3) | **1.6 (1.1-2.3)** | **1.6 (1.1-2.2)** |
| Amphetamine | 51 (0.53) | 17 (1.3) | **2.2 (1.3-3.8)** | **2.3 (1.3-4.0)** |

Note: An odds-ratio above one indicate that individuals with ADHD and coexisting ASD are more likely to receive medication and an odds-ratio below one indicate that those individuals are less likely to receive medication. Significant odds-ratios are marked as bolded.

Dexamphetamine and amphetamine required a separate application to the Swedish Medical Agency during the observation period (2005-2013).

**^#^**Adjusted for sex and birth categories in years and year of first ADHD diagnosis. **^##^**Adjusted for sex, birth categories in years, year of first ADHD diagnosis, and psychiatric comorbidities before first dispense date.

**Supplement table 5**. Sensitivity analysis after removing the 1% lowest doses and the 1% highest methylphenidate doses. Median doses of methylphenidate (mg/day) during six months intervals, comparing all individuals with ADHD with coexisting autism spectrum disorder (ASD) versus ADHD without coexisting ASD, and stratified analyses by sex, age category (adults/adolescents/children).

|  | **ADHD without coexisting ASD** | | | **ADHD with coexisting ASD** | | |  |  |
| --- | --- | --- | --- | --- | --- | --- | --- | --- |
|  | **Number of subjects (%)** | **Median**  **dose**  **(mg/day)** | **Range doses (mg)** | **Number of subjects** | **Median**  **dose**  **(mg/day)** | **Range doses (mg)** | **Z-value** | **p-value** |
| **All,**  **months** |  |  |  |  |  |  |  |  |
| **0-6** | 24891  (100) | 40.0 | 6-173 | 3369  (100) | 37.0 | 6-172 | -7.97 | < 0.0001 |
| **7-12** | 20356  (81.8) | 35.0 | 3-201 | 2729  (81.0) | 35.0 | 3-195 | -6.24 | < 0.0001 |
| **13-18** | 18142  (72.9) | 35.0 | 3-212 | 2468  (73.3) | 35.0 | 3-207 | -4.16 | < 0.0001 |
| **19-24** | 17188  (69.1) | 39.0 | 3-228 | 2345  (69.6) | 35.0 | 3-224 | -4.16 | < 0.0001 |
| **25-30** | 16122  (64.8) | 39.0 | 3-230 | 2232  (66.3) | 35.0 | 3-228 | -4.34 | 0.0016 |
| **31-36** | 15275  (61.4) | 41.0 | 3-237 | 2137  (63.4) | 38.0 | 3-237 | -3.15 | 0.0016 |
| **Adults,**  **months** |  |  |  |  |  |  |  |  |
| **0-6** | 11875  (100) | 49.3 | 6-173 | 1431  (100) | 43.4 | 6-172 | -5.35 | < 0.0001 |
| **7-12** | 9402  (79.2) | 50.4 | 3-201 | 1106  (77.3) | 44.4 | 3-195 | -3.94 | < 0.0001 |
| **13-18** | 8204  (69.1) | 50.3 | 3-212 | 989  (69.1) | 44.4 | 3-207 | -4.04 | < 0.0001 |
| **19-24** | 7733  (65.1) | 53.2 | 3-229 | 916  (64.0) | 46.4 | 3-224 | -2.49 | 0.013 |
| **25-30** | 7218  (60.8) | 53.2 | 3-230 | 867  (60.6) | 46.0 | 3-228 | -3.85 | 0.0001 |
| **31-36** | 6845  (57.6) | 53.2 | 3-237 | 816  (57.0) | 53.2 | 3-237 | -2.87 | 0.0041 |
| **Adolescents, months** |  |  |  |  |  |  |  |  |
| **0-6** | 5878  (100) | 38.4 | 6-164 | 747  (100) | 38.4 | 6-142 | -0.63 | 0.53 |
| **7-12** | 4580  (77.9) | 35.5 | 3-193 | 597  (79.9) | 35.5 | 3-124 | -0.68 | 0.49 |
| **13-18** | 3935  (66.9) | 32.9 | 3-207 | 514  (68.8) | 35.5 | 3-177 | 1.81 | 0.07 |
| **19-24** | 3614  (61.5) | 35.5 | 3-213 | 473  (63.3) | 35.5 | 3-191 | 1.99 | 0.047 |
| **25-30** | 3249  (55.3) | 35.5 | 3-204 | 443  (59.3) | 35.5 | 3-160 | 2.17 | 0.03 |
| **31-36** | 2943  (50.1) | 35.5 | 3-229 | 426  (57.0) | 35.5 | 3-181 | 2.074 | 0.038 |
| **Children,**  **months** |  |  |  |  |  |  |  |  |
| **0-6** | 7138  (100) | 31.9 | 6-169 | 1191  (100) | 29.6 | 6-112 | -3.97 | <0.0001 |
| **7-12** | 6374  (89.3) | 29.6 | 3-195 | 1026  (86.1) | 29.6 | 3-127 | -2.98 | 0.0029 |
| **13-18** | 6003  (84.1) | 29.6 | 3-186 | 965  (81.0) | 29.6 | 3-104 | -1.65 | 0.098 |
| **19-24** | 5841  (81.8) | 35.0 | 3-222 | 956  (80.3) | 31.9 | 3-149 | -3.71 | 0.0002 |
| **25-30** | 5655  (79.2) | 35.5 | 3-163 | 922  (77.4) | 32.9 | 3-160 | -2.88 | 0.004 |
| **31-36** | 5487  (76.9) | 35.5 | 3-222 | 895  (75.1) | 35.5 | 3-210 | -1.60 | 0.11 |

# Wilcoxon rank sum test used for group comparisons of doses. A negative Z-value and a p-value below 0.05 indicate that individuals with ADHD and coexisting ASD are prescribed statistically significantly lower doses as compared to individuals without coexisting ASD.

Age categories based on age at first ADHD diagnosis (adults, age ≥ 18 years, adolescents, age 13-17 years, children, age ≤ 12 years).

ADHD = attention deficit hyperactivity disorder, ASD = autism spectrum disorder, mg = milligram. Age categories defined as age at first ADHD-diagnosis.

**Supplement figure 1**. Flow-chart of study inclusion.


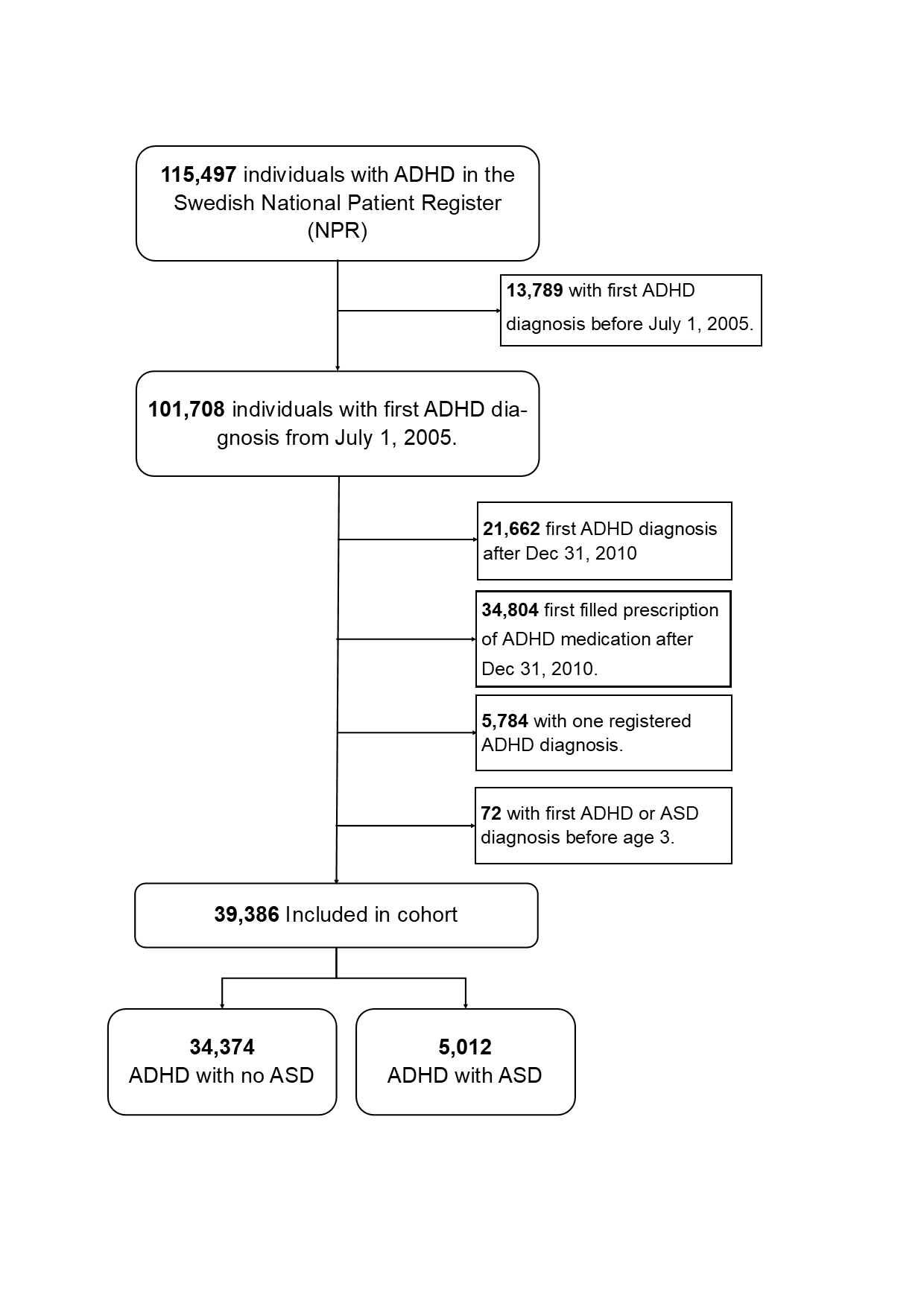


Note: ADHD = attention-deficit/hyperactivity disorder, ASD = autism spectrum disorder

**Supplement figure 2. Definitions for doses per day.** Definition for treatment start is a sequence of two dispenses within a 90-day period. Daily doses were calculated for every six months starting from the first filled prescription of the studied medication. We here give an example on how daily doses of methylphenidate was calculated during zero – six months.

**
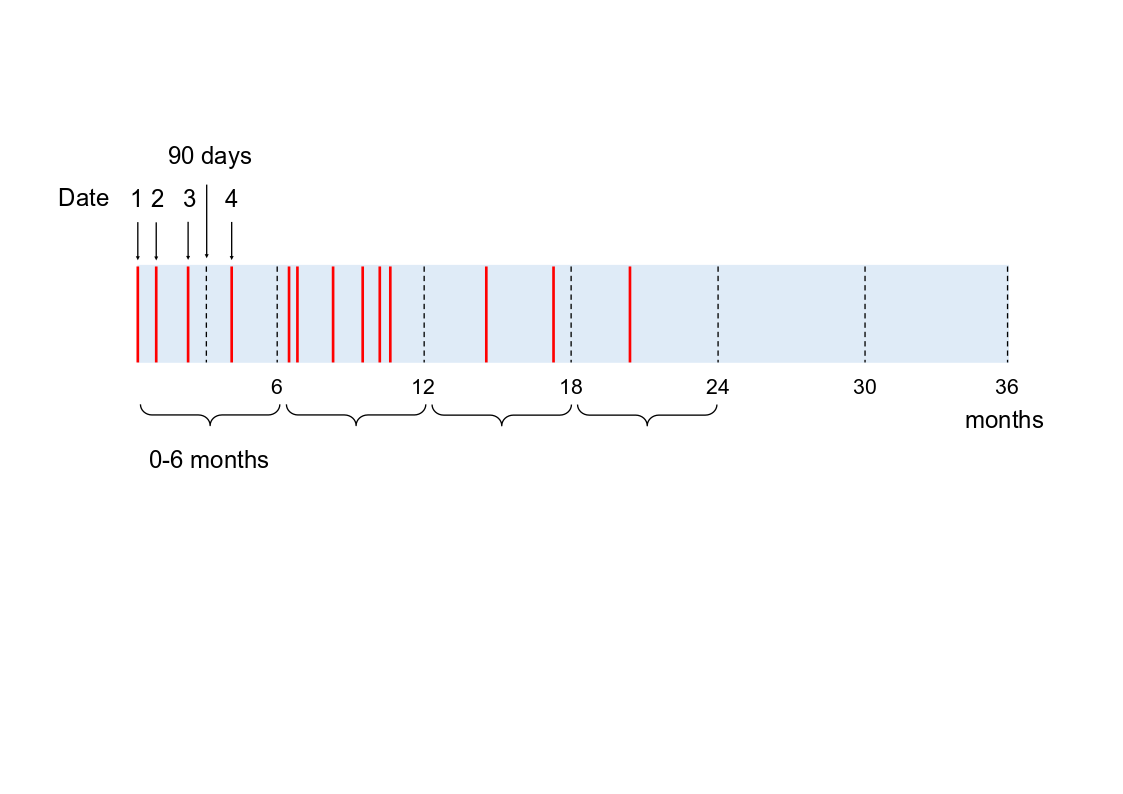
**

Example of dose calculation from register data of filled prescriptions milligram per day (mg/day), 0 to 6 months:

Date 1: Methylphenidate 18 mg, 30 pc = 540 mg

Date 2: Methylphenidate 18 mg, 30 pc = 540 mg

Date 2: Methylphenidate 36 mg, 30 pc = 1080 mg

Date 3: Methylphenidate 54 mg, 90 pc = 4860 mg

Date 4: Methylphenidate 18 mg, 90 pc = 1620 mg

Total dose: 8640 mg / 182.5 days = 47.3 mg/day

References

1. Ludvigsson JF, Otterblad-Olausson P, Pettersson BU, et al. The Swedish personal identity number: possibilities and pitfalls in healthcare and medical research. *European journal of epidemiology* 2009;24(11):659-67. doi: 10.1007/s10654-009-9350-y [published Online First: 2009/06/09]

2. Ludvigsson JF, Andersson E, Ekbom A, et al. External review and validation of the Swedish national inpatient register. *BMC public health* 2011;11:450. doi: 10.1186/1471-2458-11-450 [published Online First: 2011/06/11]

3. Ludvigsson JF, Almqvist C, Bonamy AK, et al. Registers of the Swedish total population and their use in medical research. *European journal of epidemiology* 2016;31(2):125-36. doi: 10.1007/s10654-016-0117-y [published Online First: 2016/01/16]

4. Wettermark B, Hammar N, Fored CM, et al. The new Swedish Prescribed Drug Register--opportunities for pharmacoepidemiological research and experience from the first six months. *Pharmacoepidemiology and drug safety* 2007;16(7):726-35. doi: 10.1002/pds.1294 [published Online First: 2006/08/10]
